# Trajectories of Functional Decline in Older Adults: A Latent Class Growth Curve Analysis

**DOI:** 10.1177/01939459231180365

**Published:** 2023-06-08

**Authors:** Thomas Dombrowsky

**Affiliations:** 1 University of Texas at Arlington, Arlington, TX, USA

**Keywords:** functional status, latent class growth curve modeling, functional decline trajectories, older adults

## Abstract

There have been few studies examining trajectories of functional decline among older adults in the United States using large representative databases. The purpose of this study was to describe the mean trajectory of functional decline for a representative sample of US older adults, to determine the optimal number of latent classes within that sample, and to identify key differences between the classes on select variables. Through the use of link functions, non-linear trajectories can be modeled. Three classes were identified and were named *Rapid Decline*, *Late Decline*, and *High Baseline*. The *Late Decline* Group was the most numerous and was characterized by low initial functional disability with a steep rise starting around age 85. The *Rapid Decline* Group also had low initial functional disability, but decline started around age 80. The *High Baseline* Group had high initial functional disability and less steep trajectory. Age and comorbidity were the most influential factors in functional decline. Race was statistically significant but the difference disappeared when controlling for other covariates. Sex did not significantly influence the trajectory. There were significant differences among the classes for mortality during study, initial age, initial functional status, and for several specific comorbidities: arthritis, diabetes, lung disease, and stroke.

Functional independence with regard to activities of daily living (ADL) is an important component of quality of life for older adults and for their families.^1^ Functional status is more predictive of health-related quality of life than multimorbidity among community-dwelling middle-aged and older adults.^1^ The prevalence of disability in middle-aged adults has been increasing in the United States in recent years.^2^ This is concerning. No studies could be found investigating whether the prevalence of disability is increasing for older adults, but if this trend continues it could extend to older adults. Because functional status declines with age,^3^ it is necessary to study trajectories of functional status in order to better understand the development of functional disability. A brief discussion of some of the antecedents and consequences of functional disability follows.

Functional disability is the result of a disablement process that progresses from pathology to functional consequences and is influenced by internal and external modifying factors.^2^ It is accompanied by a shrinking of social activity.^4^ Among older adults with disability, both the size of the social network and the frequency of social activity decrease. Disability onset is particularly significant for decreases in social network size. Functional disability also reduces the size of friendship groups, likelihood of social connection, and overall social connectedness.

One factor that affects the disablement process is race. Dong and colleagues found that Black non-Hispanic and Hispanic Americans experienced a more rapid transition to lower levels of functional ability than their White non-Hispanic counterparts.^5^ This tendency was somewhat lessened when socioeconomic status and health-related variables were taken into account, but was still statistically significant. Black non-Hispanic and Hispanic Americans were also less likely to successfully accommodate decreased functional status. The relationship of race to functional status is complex. Although health-related discrepancies between Black non-Hispanic and White non-Hispanic Americans are wide during middle age, they tend to narrow in old age.^6^ Functional trajectories for Black non-Hispanic and White non-Hispanic older adults are very similar until around the last year before death when they diverge, with Black non-Hispanic older adults experiencing a sharper trajectory of functional decline.

The relationship between sex and functional trajectory is also complex. One study reported that the trajectory of ADL and instrumental activities of daily living (IADL) status with age is nonlinear and differs by sex, with women having more disability at age 70 but experiencing a slower decline than men.^3^ A different study found that the ADL trajectory is linear and that sex is not a significant factor, but race is.^7^ One possible reason for the discrepancy is that the second study had older participants (mean age = 78.2, minimum age = 70 in one study^7^ vs. mean age = 63.6, minimum = 50 in the other).^3^ In the first study, when adjusted for survival time and initial upper and lower body strength, women’s disability was moderately lower than that for men, and the women’s decline trajectory was steeper.^3^ However, the women’s decline in upper and lower body strength was less pronounced, suggesting that these covariates acted differently in the case of men and women. The supplementary material for the Botoseneanu et al. article includes a chart which compares both male and female participants with starting ages of 70 and those with starting ages of 80. Both male and female participants with an initial age of 80 had much steeper declining trajectories for ADL status.^3^ Trajectory shapes as well as covariates that affect trajectories are not necessarily uniform and may depend on the ages of the participants and other factors. Studies that center on one age range may not identify covariates that are significant at other ages.

Comorbidity is another factor that affects the disablement process. Da Silva and colleagues found that abdominal obesity combined with poor grip strength doubled the annual incidence of functional disability in a cohort of older adults who were functionally independent at baseline.^8^ The number of comorbidities is a significant predictor of both baseline functional disability as well as rate of decline.^9^

The World Health Organization issued a comprehensive report on world-wide aging and health highlighting the importance of studying trajectories of functional decline among older adults.^10^ Although functional trajectories vary widely from one individual to another, even among individuals with similar medical diagnoses, the trajectories vary in predictable ways. Models of functional decline trajectories focus on the specific individual consequences of disease and are better predictors of survival and of quality of life than number of comorbidities. Knowledge of functional trajectories is useful for better understanding of individual and group outcomes.

There have been several previous studies estimating functional disability trajectories using nationally representative US datasets. Methods used include parallel process latent growth curve modeling,^11^ group based trajectory modeling,^12–14^ general estimating equations,^15^ and Markov modeling.^5^ Four of these studies used the Health and Retirement Study dataset,^11,13–15^ but two of them used National Health and Aging Trends Survey (NHATS) data.^5,12^ Four of the studies used ADL disability as the main outcome variable.^5,12–14^ The other two used physical function^15^ or physical function and memory as the outcomes.^11^

There were two studies similar to the present study: one by Martin and colleagues^13^ and another by MacNeil Vroomen and colleagues.^12^ Unlike Martin and colleagues’ study,^13^ the present study uses count data for ADLs and focuses on a range of ADL disability from no disability to disability on all ADLs. Rather than estimating class trajectories from covariates, as in the Martin et al. study, the classes are estimated from the trajectory patterns themselves and the classes are later examined for significant differences. Like the Martin study, but unlike MacNeil Vroomen and colleagues’ study,^12^ the present study estimates trajectories for the entire age range of the participants.

## Purpose

Few studies have described trajectories of functional decline using population-based large datasets of US older adults. The purpose of the present study is to describe typical trajectories of functional decline in the US population aged 65 years and older. Specific aims are to identify the average trajectory of functional decline in a representative sample of US older adults, to identify the optimal number of latent classes in that sample, and to identify specific differences among those classes.

## Methods

Growth curve models evaluate changes in an outcome variable over time, combining individual and group trajectories, as well as capturing factors that influence those trajectories.^16,17^ By combining cohorts at different stages of development, longitudinal studies can be extended to model time frames longer than the actual duration of the study. The techniques of structural equation modeling are used. Unconditioned models simply estimate the average trajectory of the whole sample. Covariates thought to be related to the trajectory can be added to the unconditioned model and their effect can then be measured. If the sample is not homogeneous for trajectory, latent class models can be used to identify the different trajectories in the sample. The present study includes an unconditioned model, a covariate model, and a latent class model.

Latent class analysis extends the growth curve method and incorporates the techniques of factor analysis to identify different trajectories existing within a group.^17^ This is done by postulating the existence of two or more mutually exclusive classes. Each sample member is then assigned a class using maximum likelihood methods. Trajectories for each of the resulting classes can then be examined, and the members of the classes can be examined for commonalities. Latent class analysis makes the assumption that some of the heterogeneity in a sample can be explained by dividing the sample into latent classes.^18^ The analysis does not return the classes themselves, but rather probabilities of being in a specific class. Each participant has some probability of being in each of the classes identified but is assigned to the class for which they have the highest probability. The quality of the total model can be evaluated by determining how distinctly the classes are separated.

### Sample

The NHATS is an ongoing survey using a nationally representative stratified random sample of Medicare recipients.^19^ The survey began in 2011. Currently 11 annual rounds of data are available, but only rounds one through 10 were used in the present study. Data collection for these rounds occurred between 2011 and 2020. There was one replenishment in 2015, so all NHATS participants had their initial year either in 2011 or 2015. Total numbers were 8,245 in the initial cohort and 4,182 in the 2015 replenishment, for a total of 12,427.^20^ Since non-Hispanic African Americans and the oldest old were over-represented in the original sample, the sample for the present study was obtained by randomly selecting 3,000 participants from the original 2011 cohort taking account their NHATS sample weights. Of these 3,000 participants, 110 had to be excluded for lack of data regarding their functional status. The only data available on the excluded participants were race, gender, age, and residential status. The 110 excluded participants were older (mean 83.7 years vs. 76.4 years, *p* < 0.001). Proportion z testing showed that differences in race and gender between the excluded participants and the rest of the sample were not statistically significant. The vast majority of the excluded participants (92.7%) lived in nursing homes. There were 2,890 participants in the final sample. All 2,890 participants were used in the unconditioned model and the class model, but only 2,856 participants were used in the covariate model due to 34 participants with missing data concerning primary race. Since the data used for this study was already deidentified, the University of Texas at Arlington Institutional Review Board waived approval for this study.

### Measurements

In the NHATS survey, participants were asked about whether or not they needed assistance with eating, bathing, dressing, and going to the toilet.^19^ For the present study a dichotomous variable was constructed for each of these activities. Participants who used assistive devices were scored as zero, as long as they reported always doing the activity without assistance. These scores were then summed to create a modified Katz score ranging from zero (no assistance with any activity) to four (assistance with all activities).

The following demographic data were extracted and used in this study: birth year and month, year of death, sex, race, Hispanic ethnicity, and marital status.^19^ The following diagnoses were also extracted: heart attack, heart disease, hypertension, arthritis, osteoporosis, diabetes, lung disease, stroke, dementia, and cancer. Participants were asked whether they had ever been diagnosed with these conditions by a physician. The demographic data and diagnoses were taken from the first round in which a participant was enrolled. There was also a variable for death during the course of the study.

Age was modified by subtracting 65 from the chronological age, so that the youngest participants had an adjusted age of zero. This was done to facilitate data analysis. Race and ethnicity were categorized as either White, Black, other, or Hispanic, and was taken from the primary race listed in the NHATS data. Sex was either male or female. Comorbidities were added to produce a numerical score ranging 1–10 which was used in the growth curve models, but only individual comorbidities were used in the multinomial logistic regression model. Comorbidities and modified Katz levels were calculated for all rounds. The smoking variable was compiled from two questions in the first round asking about whether the person ever smoked and whether they smoke now.

For the social network variable, participants were asked to name up to five people that they regularly speak to about things that are important to them.^19^ The number of people named was summed. In addition, three first-round questions about the community were used asking whether people in the community know each other well, whether they are willing to help each other, and whether they can be trusted. Possible answers were *agree a lot*, *agree a little*, and *do not agree*. Participation variables were generated from responses to questions about whether they had participated in the following activities over the last month: visiting family and friends; attending religious services; attending clubs, classes, or other activities; going out for enjoyment; and engaging in vigorous activity. The participation variables were all dichotomous with *yes* and *no* being the only responses.^19^

### Data Analysis

Data analysis was done using the R Language and Environment for Statistical Computing^21^ and the lcmm package.^22^ Because growth models contain repeated measures of the same individuals, mixed modeling techniques are incorporated into the analysis. Most growth curve modeling software focuses on fitting linear models, but Proust-Lima and colleagues have developed software that can fit binomial, ordinal, or Poisson models. In order to estimate nonlinear models the software makes use of link functions. The link functions connect the actual measurements in the data with a hypothesized latent variable that is continuous and normally distributed. Available link functions include binomial, splines, and threshold functions. The splines link function (which was used in the present study) produces a normalized transformation of the dependent variable. With splines, trajectories do not need to be linear or even curvilinear. An intercept is estimated, representing the initial value of the trajectory, and subsequent nodes for each measurement point are also estimated.

Models can be evaluated by starting with a one-class model and successively adding classes so long as the added class improves model fit as evidenced by lower Bayesian Information Criterion score.^18^ Other considerations include model interpretability and entropy scores. Entropy levels indicate how well classes are assigned in the model, and should be between 0.8 and 1.0. Another criterion is posterior probability of class assignment. Each individual in the model has a specific probability of being assigned to each of the classes. Individuals are finally assigned to the class for which they have the highest probability. Posterior probabilities are calculated by averaging the probabilities of each individual who is finally assigned to the class. Ideally, posterior probabilities should be 0.90 or higher, but posterior probabilities greater than 0.80 are acceptable.

Exploratory models were fit using linear, binomial, threshold, and splines link functions. The following models were fit: an unconditioned model, representing a mean trajectory using only age and age squared; a covariate model using age, age squared, race/ethnicity, gender, and comorbidity as covariates; and class models based on the unconditioned model to identify heterogeneous trajectories. Once classes were identified, a multinomial logistic regression analysis was performed comparing each of the classes with the rest of the sample for demographic, disease, community, and activity variables using the nnet package in R.^23^ This was done to identify significant predictors of class membership from the variables available and thus highlight differences between the classes while controlling for covariates. Since the regression could only be done using complete data, the regression was done on existing data and was repeated using an imputed dataset. Missing data points were imputed using the mice package in R with predictive mean matching.^24^ This method replaces missing values with values randomly chosen from the complete observations in the dataset that most closely match the observation with the missing data point. There was little difference between the imputed model and the model with missing data, so only the model with actual observed data is reported here.

## Results

Of the 2,890 participants in the final sample, 56.9% were women and 43.1% were men. The mean age for all participants in 2011 (the beginning of the study) was 75.9 years. The distribution of the sample by race and ethnicity was 78.8% White non-Hispanic, 10.8% Black non-Hispanic, and 6.9% Hispanic. The mean Katz scores by race/ethnicity in the first year were 0.41 for White non-Hispanics, 0.53 for Black non-Hispanics, and 0.70 for Hispanics. Pairwise Kruskal tests showed that mean initial Katz score was lower for White non-Hispanics than for Hispanics (*p* = .003). For men of all races the initial mean Katz was 0.39 and for women it was 0.48 (*p* = .004).

### Models

**Figure 1.**
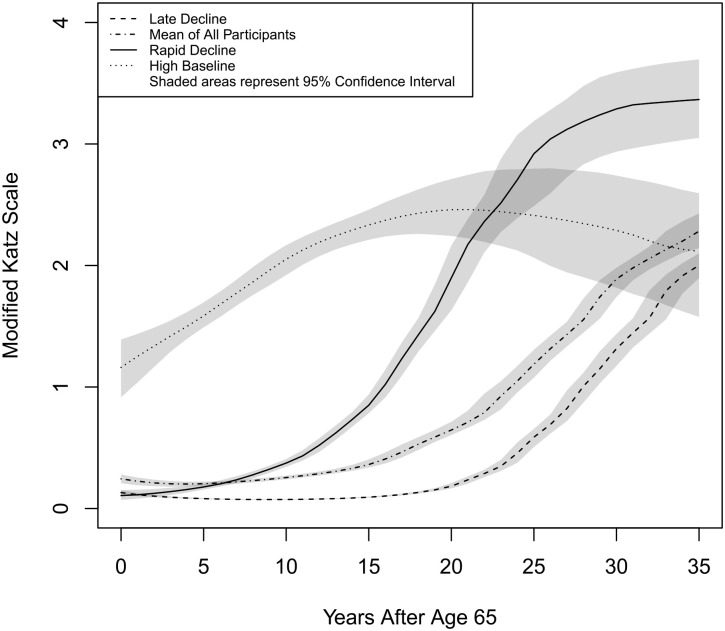
Functional Trajectories by Class.

**Figure 2.**
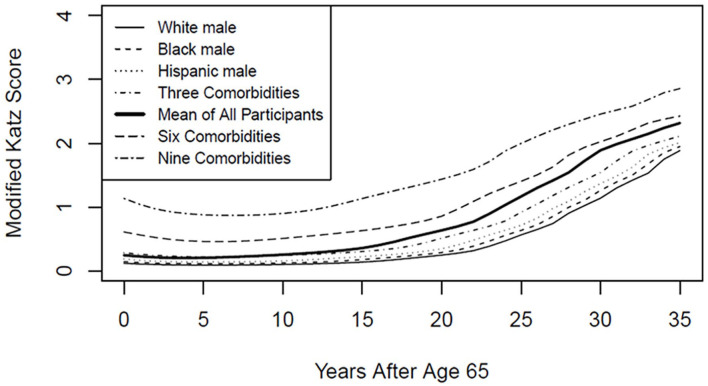
Trajectories by Selected Covariates.

Competing models were selected based on decreased Bayesian Information Coefficient score. The unconditioned model with the lowest Bayesian Information Coefficient used a five-node equidistant splines link function and had age and age squared both for fixed effects and random effects and the modified Katz score as the outcome variable. This unconditioned model served as the basis for the covariate model and the class models. The best fitting class model was the three-class model. Fitness measures for the models are given in Table 1, and coefficients in Table 2. The trajectory for the unconditioned model is shown in Figures 1 and 2 as the “Mean of All Participants” trajectory.

**Table 1.** Fit Measures for Models.

	Log Likelihood				Mean Posterior Probabilities for Class Membership			
Model		AIC	BIC	Entropy	Class 1	Class 2	Class 3	Class 4
Unconditioned beta *n* = 2,890	–6,815.322	13,654.644	13,726.272	1				
Unconditioned linear *n* = 2,890	–17,704.730	35,429.459	35,489.150	1				
Covariate splines *n* = 2,856	–2,229.074	4,498.147	4,617.291	1				
Unconditioned splines *n* = 2,890	–2,693.917	5,417.835	5,507.370	1				
2 group splines *n* = 2,890	–2,093.235	4,224.469	4,337.880	0.875	0.975	0.903		
3 group splines *n* = 2,890	–1,835.646	3,717.292	3,854.579	0.806	0.786	0.940	0.863	
4 group splines *n* = 2,890	–1,835.646	3,725.293	3,886.456	0.470	NaN	0.559	0.856	0.725

AIC = Aikake Information Criterion, BIC = Bayesian Information Criterion, NaN = not a number.

Note: The four class model converged with a class that had no members (class 1).

**Table 2.** Model Covariates.

Unconditioned Model (N = 2,890)									
	Intercept	Adjusted Age	Adjusted Age^2^	Spline 1	Spline 2	Spline 3	Spline 4	Spline 5	Spline 6
	0.000	−0.053 (0.009) < 0.001***	0.004 (0.0003)< 0.001***	−0.670(0.052) < 0.001***	1.627 (0.006) < 0.001***	0.003 (0.001) 0.999	1.027(0.012)< 0.001***	–0.129 (0.187) 0.490	0.871 (0.025) < 0.001***
Covariate Model (N = 2,856)									
	Intercept	Adjusted Age	Adjusted Age^2^	Spline 1	Spline 2	Spline 3	Spline 4	Spline 5	Spline 6
	0.000	−0.074(0.004)< 0.001***	0.004(.0003)< 0.001	−0.127 (0.058) 0.029*	1.635 (0.006) < 0.001***	0.0003 (0.003) 0.926	1.032 (0.012) < 0.001***	0.160 (0.132) 0.225	0.877(0.023)< 0.001***
						Female	Black	Other Race	Hispanic
						–0.008 (0.039) 0.843	0.197 (0.069) 0.004**	–0.120 (0.117) 0.308	0.412 (0.086) < 0.001***
Class Model (N = 2,890)									
Class	Intercept	Adjusted Age	Adjusted Age^2^	Spline 1	Spline 2	Spline 3	Spline 4	Spline 5	Spline 6
RD	0.000	0.084 (0.028) 0.003**	0.003 (0.001) 0.005**	−0.282 (0.161) 0.079	1.631 (0.006) < 0.001***	0.000 (0.009) 1.000	1.034 (0.012) < 0.001***	–0.219 (0.098) 0.025*	0.854 (0.023) < 0.001***
LD	0.172 (0.178) 0.335	−0.085 (0.008) < 0.001***	0.005 (0.0003) < 0.001***	Same for all classes					
HB	2.325 (0.196) < 0.001***	0.106 (0.024) < 0.001***	−0.002 (0.001) 0.046*						

RD = Rapid Decline Group, LD = Late Decline Group, HB = High Baseline Group,

* *p* < .05. ** *p* < 01, *** *p* < .001.

Note: Coefficients followed by standard deviations (in parentheses) and probabilities. Intercepts were fixed at zero for the one class models and for the first class of the class model.

The covariate model adjusted for gender, race, and comorbidity. In this model, age, Black non-Hispanic race, Hispanic ethnicity, and comorbidity were significant predictors of functional status, but sex and other race were not. Most likely this is due to the small numbers and heterogeneity of the other race group. This model was used to create the trajectories pictured in Figure 2.

The latent class model yielded three classes. The classes were named Rapid Decline Group, Late Decline Group, and High Baseline Group. A four-class model was also fit, but it had a higher BIC score than the three-class model as well as a worse entropy score than the three-class model (0.470 vs. 0.806). Entropy levels less than 0.8 suggest that the classes may be poorly differentiated.^18^ See Table 1 for comparisons of models.

In the three-class model for the present study, the posterior probabilities were: Rapid Decline = 0.786, Late Decline = 0.940, and High Baseline = 0.863. The three-class model only had one class with a posterior probability score less than 0.8 (the Rapid Decline Group), but the four-class model had two such classes. Coefficients of the three-class model are given in Table 2, and trajectories for each class are given in Figure 1.

### Comparison of Classes

The three-class splines model divided the sample into three latent classes. Sample characteristics as well as characteristics of the differences among the classes are summarized in Table 3. The Rapid Decline Group contains about 10.52% of the participants and is characterized by low functional disability at age 65. However, members of this group began to decline almost immediately and their curve became steeper beginning around age 80. The High Baseline Group contains about 10.83% of the participants. Members of this class had higher functional disability at age 65 than those of the other classes and had a slow steady increase in functional disability until around age 85, at which point it plateaued. The Late Decline Group comprised about 78.65% of the participants. Participants in this class had low levels of functional disability at age 65. They began their disability trajectory around age 85 and had a fairly steep increase thereafter.

**Table 3.** Distribution of Main Variables by Class With Regression Results.

	Group Breakdown				Model for Class Membership (*n* = 2,496)					
Variable	ED	LD	HB	All	ED			HB		
	n(%)/M ± SD				OR	95% CI	*p*	OR	95% CI	*p*
Female (reference male) *n* = 2,890	186 (61.2)	1276 (56.1)	182 (58.1)	1644 (56.9)	1.204	(0.849–1.707)	.298	1.002	(0.621–1.618)	.993
Black (reference White) *n* = 2,867	29 (9.6)	225 (10.0)	55 (17.6)	309 (10.8)	0.797	(0.491–1.295)	.360	1.248	(0.682–2.283)	.515
Other race	11 (3.6)	81 (3.6)	7 (2.2)	99 (3.5)	0.858	(0.374–1.972)	.719	0.404	(0.090–1.816)	.254
Hispanic any race	24 (7.9)	140 (6.2)	35 (11.2)	199 (6.9)	0.838	(0.453–1.548)	.572	1.076	(0.480–2.411)	.870
Living with partner (reference married) *n* = 2,889	10 (3.3)	57 (2.5)	12 (3.8)	79 (2.7)	1.521	(0.691–3.348)	.297	0.743	(0.240–2.298)	.613
Separated	9 (3.0)	28 (1.2)	8 (2.6)	45 (1.6)	2.098	(0.779–5.652)	.143	0.983	(0.199–4.866)	.984
Divorced	37 (12.2)	241 (10.6)	39 (12.5)	317 (11.0)	1.244	(0.783–1.976)	.355	1.001	(0.524–1.912)	.998
Widowed	99 (32.7)	642 (28.2)	91 (29.1)	832 (28.8)	0.917	(0.625–1.345)	.657	0.838	(0.494–1.421)	.538
Never married	12 (4.0)	83 (3.7)	13 (4.2)	108 (3.7)	1.099	(0.517–2.336)	.807	0.532	(0.173–1.633)	.293
Died (reference survived) *n* = 2,890	108 (35.5)	486 (21.4)	130 (41.5)	724 (25.1)	2.131	(1.481–3.067)	<.001***	3.109	(1.896–5.098)	<.001***
People know each other well: agree a little (reference disagree) *n* = 2,838	114 (38.1)	898 (40.2)	115 (37.7)	1127 (39.7)	1.289	(0.823–2.018)	.268	1.552	(0.814–2.961)	.210
People know each other well: agree a lot	125 (41.8)	894 (40.0)	129 (42.3)	1148 (40.5)	1.173	(0.713–1.931)	.529	1.825	(0.901–3.694)	.118
People can be trusted: agree a little (reference disagree) *n* = 2,761	84 (28.7)	596 (27.4)	97 (32.9)	777 (28.1)	0.711	(0.407–1.242)	.231	0.614	(0.296–1.274)	.213
People can be trusted: agree a lot	173 (59.0)	1385 (63.7)	150 (50.8)	1708 (61.9)	0.597	(0.338–1.052)	.074	0.563	(0.268–1.179)	.151
People willing to help: agree a little (reference disagree) *n* = 2,807	103 (34.9)	762 (34.6)	111 (36.2)	976 (34.8)	1.511	(0.809–2.822)	.196	1.059	(0.477–2.352)	.895
People willing to help: agree a lot	162 (54.9)	1245 (56.5)	153 (49.8)	1560 (55.6)	1.929	(0.982–3.791)	.057	1.206	(0.498–2.918)	.699
Visit friends and family: yes (reference no) *n* = 2,886	257 (84.5)	2009 (88.5)	239 (76.8)	2505 (86.8)	1.086	(0.689–1.712)	.721	1.396	(0.738–2.639)	.318
Attend religious services: yes (reference no) *n* = 2,887	162 (53.3)	1343 (59.1)	164 (52.7)	1669 (57.8)	1.100	(0.807–1.500)	.546	1.300	(0.840–2.011)	.271
Participate in clubs classes & other group activities: yes (reference no) *n* = 2,888	94 (30.9)	873 (38.4)	72 (23.1)	1039 (36.0)	1.306	(0.935–1.824)	.118	1.396	(0.861–2.262)	.213
Go out for enjoyment: yes (reference no) *n* = 2,887	214 (70.4)	1820 (80.1)	186 (59.6)	2220 (76.9)	0.708	(0.496–1.012)	.058	0.649	(0.403–1.045)	.087
Work for pay: yes (reference no) *n* = 2,886	34 (11.2)	422 (18.6)	24 (7.7)	480 (16.6)	0.729	(0.471–1.127)	.155	0.746	(0.405–1.372)	.407
Do volunteer work: yes (reference no) *n* = 2,888	48 (15.8)	591 (26.0)	36 (11.5)	675 (23.4)	0.709	(0.480–1.048)	.085	0.773	(0.446–1.339)	.410
Engage in vigorous activity: yes (reference no) *n* = 2,887	90 (29.6)	923 (40.6)	64 (20.5)	1077 (37.3)	0.861	(0.622–1.192)	.368	0.815	(0.513–1.294)	.426
Heart Attack: yes (reference no) *n* = 2,885	78 (16.9)	397 (12.2)	96 (24.0)	571 (14.0)	0.857	(0.551–1.335)	.496	0.967	(0.543–1.720)	.913
Heart disease: yes (reference no) *n* = 2,883	117 (22.4)	582 (16.6)	113 (28.0)	812 (18.5)	1.133	(0.774–1.661)	.520	1.283	(0.769–2.139)	.369
Hypertension: yes (reference no) *n* = 2,886	246 (71.0)	1602 (62.3)	255 (77.6)	2103 (64.8)	1.238	(0.896–1.711)	.196	1.191	(0.752–1.888)	.494
Arthritis: yes (reference no) *n* = 2,883	256 (67.7)	1469 (51.3)	250 (69.8)	1975 (55.0)	1.540	(1.129–2.100)	.006**	1.425	(0.922–2.202)	.145
Osteoporosis: yes (reference no) *n* = 2,878	129 (24.2)	684 (20.3)	121 (28.9)	934 (21.6)	0.763	(0.525–1.109)	.157	0.734	(0.445–1.210)	.254
Diabetes: yes (reference no) *n* = 2,889	118 (33.9)	591 (21.6)	137 (40.7)	846 (25.0)	1.476	(1.072–2.033)	.017*	1.640	(1.066–2.524)	.035 *
Lung disease: yes (reference no) *n* = 2,886	104 (24.1)	457 (13.0)	102 (25.6)	663 (15.5)	1.631	(1.135–2.345)	.008**	1.422	(0.867–2.331)	.183
Stroke: yes (reference no) *n* = 2,888	75 (14.1)	286 (7.5)	92 (23.7)	453 (10.0)	1.455	(0.910–2.325)	.117	2.324	(1.302–4.150)	.006 **
Dementia: yes (reference no) *n* = 2,887	88 (10.9)	206 (2.8)	74 (12.8)	368 (4.7)	2.036	(0.914–4.531)	.082	1.326	(0.416–4.225)	.609
Cancer: yes (reference no) *n* = 2,887	113 (24.7)	790 (26.6)	107 (25.9)	1010 (26.3)	0.736	(0.523–1.036)	.079	0.782	(0.490–1.247)	.340
Quit smoking (reference never smoker) *n* = 2,890	146 (48.0)	988 (43.5)	136 (43.5)	1270 (43.9)	1.314	(0.958–1.802)	.091	1.082	(0.690–1.695)	.750
Current smoker	25 (8.2)	184 (8.1)	40 (12.8)	249 (8.6)	0.916	(0.522–1.607)	.759	1.456	(0.733–2.893)	.337
Katz Score in 2011 *n* = 2,873	0.852 ± 1.100	0.167 ± 0.565	2.062 ± 1.238	0.441 ± 0.948	3.834	(3.065–4.796)	<.001***	13.143	(9.902–17.446)	<.001***
Age in 2011 *n* = 2,890	76.161 ± 6.711	76.077 ± 7.811	73.934 ± 5.949	75.854 ± 7.548	0.919	(0.896–0.944)	<.001***	0.799	(0.766–0.833)	<.001***
Social network *n* = 2,716	1.830 ± 1.174	1.930 ± 1.293	1.958 ± 1.379	1.923 ± 1.290	0.926	(0.821–1.044)	.207	1.044	(0.888–1.227)	.630
Comorbidity in 2011 *n* = 2,852	3.095 ± 1.726	2.338 ± 1.509	3.567 ± 1.630	2.549 ± 1.602	(not included in multinomial logistic regression model)					

ED = Early Decline Group, LD = Late Decline Group, HB = High Baseline Group, * *p* < .05, ** *p* < .01, *** *p* < .001.

Note: Sample numbers in the left column are for proportions and means only.

The Late Decline Group, the most numerous class, was characterized by a lower prevalence of diabetes (21.6% vs. 33.9%, *p* = .017; 21.6% vs. 40.7%, *p* = .035) and of death during the course of the study (21.4% vs. 35.5%, *p* < .001; 21.4% vs. 41.5%, *p* < .001). The Late Decline Group had lower prevalence of arthritis (51.3% vs. 67.7%, *p* = .006) and lung disease (13.0% vs. 24.1%, *p* = .008) than the Early Decline Group, and had lower prevalence of stroke than the High Baseline Group (7.5% vs. 23.7%, *p* = .006). Initial age was higher for the Late Decline Group than for the High Baseline Group (76.08 vs. 73.93, *p* < .001), and initial Katz Score was lower than the other two groups (0.17 vs. 0.85, *p* < .001; 0.17 vs. 2.06, *p* < .001). There were no significant differences among the groups for the demographic, community, or activity variables. There were no significant differences for heart attack, heart disease, hypertension, osteoporosis, dementia, cancer, smoking, or size of social network. Although there were no significant differences for race among the groups in the multinomial logistic regression model, when race was considered alone there were significant differences. A chi-square test for race by class resulted in a chi-square value of 31.58 with 6 degrees of freedom (*p* < .001). A similar test for sex was not significant, with a chi-square value of 3.01 with 2 degrees of freedom (*p* = .222).

### Trajectories

In the covariate model, comorbidities and age had the largest influence, overriding effects of gender and age. This can be clearly seen in Figure 2. The greatest difference for race is between White non-Hispanic and Hispanic participants, yet on the graph the lines for these two demographic groups can barely be distinguished. In contrast, the lines between the varying levels of initial comorbidity can easily be distinguished. In contrast to Figure 1, confidence intervals were not given in Figure 2 because there would have been too much overlap. Trajectories for White non-Hispanic, Black non-Hispanic, and Hispanic ethnicity were calculated based on a male with no comorbidity at Round 1. Zero comorbidity is unusual, as can be seen by the fact that all three ethnicity curves are lower than the mean curve for all participants. The comorbidity trajectories were calculated for a White non-Hispanic male. Female trajectories for each ethnicity were so close to the male trajectories as to be practically indistinguishable.

## Discussion

There were no significant differences by sex among groups. This is a divergence between the present study and other studies which did find sex to be significant in their growth models.^3,9^ Chen and colleagues found that being female was associated with faster growth in disability but not with higher disability at outset.^9^ The dependent variable for the Chen and colleagues study was general functional disability, a latent variable with ADLs, IADLs, and functional limitations as indicators. .

While not statistically significant in the multinomial logistic regression model, non-Hispanic African American participants were over-represented in the Rapid Decline Group and the High Baseline Group. Taylor and colleagues specifically studied trajectories of ADL limitations in the years prior to death and found that while racial disparities in functional status generally diminish in late old age, the trajectories diverge in the last two years before death with the curve becoming steeper for non-Hispanic African Americans.^6^ This is also consistent with the study by Kail and colleagues who found that while the risk of functional limitation was similar among White non-Hispanic, African American non-Hispanic, and Hispanic older adults when they had no comorbidities, the curves for both African Americans non-Hispanics and Hispanics became steeper when comorbidities were present.^25^

One possible reason for racial disparities in functional status is neighborhood segregation. Frailty in older adults is positively associated with living in neighborhoods with a higher proportion of African Americans.^26^ However, living in areas with high proportion of African Americans could be a proxy for many other cultural, economic, and environmental factors. This area of research remains underexplored.

Zamudio-Rodrígues and colleagues compare and contrast frailty and disability.^27^ Frailty represents a state of increased vulnerability to stressors. It involves weakness, exhaustion, weight loss, slowness, and low physical activity, and is often operationally defined as the presence of at least three of those characteristics.^28^ Disability is difficulty performing ADLs or IADLs or difficulty with mobility.^27,28^ The researchers proposed a four-stage model of functional disablement where there is a progression from frailty to IADL disability and finally ADL disability.^27^ They tested this model using Cox regression and obtained results that supported the proposed model.

The Caldwell et al. study used similar neighborhood questions to the ones used in the present study and grouped them into a variable that they called social cohesion.^26^ Neighborhoods that are socially cohesive may provide health benefits for their residents by promoting healthy behavior, encouraging mutual trust, and building stronger social networks. The researchers found a significant negative relationship between social cohesion and frailty. They also found positive relationships between frailty and physical disorder (as rated by the interviewer), neighborhood instability, and higher proportions of African American residents. In the present study none of the neighborhood variables are statistically significant when adjusted for covariates. One explanation for the discrepancy is that the Caldwell and colleagues study used frailty as the dependent variable. The present study focuses on ADL dependency. If the Zamudio-Rodrígues model mentioned above is true, ADL dependency is a later stage of the disablement process than frailty.^27^

Comorbidity was the covariate that had the most influence on functional status in the covariate one-class model of the present study. Chen and colleagues used a population based Taiwanese cohort of older adults to study functional decline in a one-class growth model.^9^ They found that number of comorbidities significantly increased baseline functional disability and also resulted in a steeper growth trajectory. Total comorbidity could not be included in the regression model for the present study due to issues of multicollinearity, but there were also significant class differences in incidence of specific comorbidities, with three of the 10 comorbidities being statistically predictive of less likelihood of membership in the Late Decline Group. While the present study did find differences in baseline functional status on the basis of initial comorbidity (see Figure 2), it did not find notable differences in steepness of growth. This could be to differences in the specific measurements included in the present study and that of Chen and colleagues.

Fingerman et al. suggest that limitations in functional status themselves may involve a positive feedback loop whereby limited activity leads to greater engagement with unhealthy activity with a final result of even greater functional disability.^29^ The older adults with functional disability had greater sedentary behavior, less physical activity, and less activity outside the home. In the current study the differences in participation in discretionary activities all followed expected patterns for classes (highest participation in Late Decline, moderate in Rapid Decline, and lowest in High Baseline), but these differences were not statistically significant in the regression model.

### Limitations

One limitation of this study is attrition of the sample. Attrition occurred either due to deaths in the cohort or due to failure to respond to subsequent rounds of the survey. It cannot be assumed that non-respondents in either of these situations are similar to respondents. The Rapid Decline class had less than optimal differentiation in the class model as evidenced by a post probability score of 0.786.

The present study uses adjusted age as the time variable rather than year of data collection. Participants enrolled in the study in either year one or year five and were followed until they died or were lost to follow-up. By using age as the time variable rather than year of the round of data collection, the trajectory for the model can be extended beyond the number of years of actual data collection. However, this extension does not come without cost. Finkel and Ernsth Bravell point out that there may be generational differences between cohorts which can affect the dependent variable.^30^

Only a subset of the covariates that could potentially impact functional status were used. For example, this study lacks variables related to educational level, profession, and socioeconomic status. There are variables related to educational level and socioeconomic status in the NHATS dataset, but they were not in a form amenable to use in this study. This limitation is important in that access to healthcare is closely related to socioeconomic conditions. Another limitation is that most of the data used in this study is self-report data. Older adults in nursing homes consistently overestimate their functional abilities compared to proxy ratings and ratings by researchers, especially when they have impaired cognitive function.^31^ A study of community-dwelling older adults found that self-estimates of ADL ability were generally better than self-estimates of abilities such as walking six blocks, and that the estimates were more accurate regarding functional abilities as opposed to disabilities.^32^ High levels of depression are associated with underestimation of functional abilities.^33^ Both overestimation and underestimation are thus potential problems with self-report data.

## Conclusion

The present study has several implications for clinical practice and for future research. Estimates of mean trajectory can give patients, their families, and healthcare providers an idea of what to expect when they encounter older adults who have entered a pattern of functional decline. As seen in Figure 2, the classes in the present model were most clearly distinguished between the ages of around 75–80. Models can also identify factors that enhance or diminish functional decline. The models presented here, together with other similar models, can be used to further a better understanding of the disablement process. Future studies should focus on expanding the context of disablement trajectories by using longer time spans. No doubt there are important precursors in middle age that could be predictive of later functional decline. This is especially important with regard to the High Baseline class, because the trajectory of functional decline for that group actually begins before age 65, and so the beginning phase of that trajectory was not captured in the present study. Future work should focus on ways of identifying which trajectory a given individual is on as well as refining the predictive capabilities of the trajectories. This research can also be expanded by incorporating other indicators of functional decline such as IADL and performance-based indicators of functional status.

Trajectories of functional status of older adults are not linear but are curvilinear and are better represented using splines. Three distinct trajectories were identified in this study; rapid decline, high baseline, and late decline. Significant differences in the characteristics of each of these groups were identified. Comorbidities are the most important factor in the disablement process, but race is also important. There is still work to be done in explaining the racial influence on disablement.
